# Possession of Injectable Epinephrine Among Children with Parent-Reported Food Allergies in Saudi Arabia

**DOI:** 10.3390/jcm14155274

**Published:** 2025-07-25

**Authors:** Amer Khojah, Ameera Bukhari, Ibrahim Alibrahim, Maria AlSulami, Turki Alotaibi, Ruba Alotaibi, Elaf Bahareth, Inam Abulreish, Sumayyah Alsuruji, Raghad Rajab, Loie Goronfolah, Mohammad Binhussein, Adeeb Bulkhi, Saddiq Habiballah, Imad Khojah

**Affiliations:** 1 Department of Pediatrics, College of Medicine, Umm Al-Qura University, Makkah 21955, Saudi Arabia; 2 College of Science, Taif University, Taif 21944, Saudi Arabia; 3 College of Medicine, Umm Al-Qura University, Makkah 21955, Saudi Arabia; 4 King Abdullah International Medical Research Center, King Saud Bin Abdulaziz University for Health Sciences, Jeddah 22384, Saudi Arabia; 5 Department of Internal Medicine, College of Medicine, Umm Al-Qura University, Makkah 21955, Saudi Arabia; 6 Faculty of Medicine, King Abdulaziz University, Jeddah 21589, Saudi Arabia

**Keywords:** food allergy, epinephrine auto-injector, epinephrine, parental education, Saudi Arabia

## Abstract

**Background/Objectives**: A food allergy (FA) is an immune-mediated hypersensitivity reaction to specific food. FA reactions vary from mild to life-threatening anaphylaxis. Despite the effectiveness of epinephrine auto-injectors (EAIs), barriers such as lack of knowledge, limited access, and fear of needles hinder their use. This study explores EAI possession among children with parent-reported food allergies in Saudi Arabia. **Methods**: A cross-sectional study conducted from October 2023 to February 2024 included 296 parents of children with reported food allergies under the age of 18. Data were collected through a validated self-administered questionnaire. **Results**: Among 2102 respondents, 296 (14.1%) reported having a child with a food allergy. Most respondents were female (70%), with asthma being the most common comorbidity (26%). Common allergens included eggs, tree nuts, peanuts, milk, and sesame. Only 23.3% of children had an EAI. Higher EAI possession was associated with parental education, maternal allergy history, and access to specialist care. **Conclusions**: EAI possession among Saudi children with food allergies is suboptimal. Targeted educational interventions, increased access to allergists, and comprehensive management plans are essential to improve preparedness for anaphylaxis.

## 1. Introduction

A food allergy (FA) is a reproducible immune-mediated hypersensitivity reaction to specific foods [1]. Although FAs can affect individuals of all ages, studies consistently show a higher prevalence in young children, with some evidence indicating an increasing prevalence over time [2,3,4]. The perceived prevalence of pediatric FAs based on parents’ reports ranges from 6% to 16%, with Saudi Arabia among the countries on the higher end of this range [5,6,7,8,9]. FA reactions can vary from mild local reactions to severe and potentially life-threatening anaphylactic reactions that require immediate medical intervention [10]. Fortunately, injectable epinephrine is a very effective treatment for food-induced anaphylaxis, reversing the reaction and potentially saving the patient’s life [11,12,13]. Epinephrine has a rapid onset of action and leads to vasoconstriction, increasing blood pressure and stimulating the heart. It also promotes airway smooth muscle relaxation and bronchodilation [14,15]. Autoinjector devices have made it easy and safe to deliver epinephrine intramuscularly by the parents during emergencies [14,15,16]. Furthermore, epinephrine auto-injectors (EAIs) can be stored safely at room temperature for extended periods of time [17]. Despite all these advances, there are some barriers to EAI utilization, including lack of knowledge, affordability, and fear of needles [18,19]. Delayed administration of injectable epinephrine can result in increased mortality rates for food-allergic patients [20]. Understanding the possession rate of EAIs is essential for public health planning and education strategies [21]. It provides insights into how well prepared families are at manage anaphylactic emergencies before seeking healthcare at a hospital. This study aims to explore the rate of EAI possession among children with parent-reported food allergies and identify factors influencing its use.

## 2. Materials and Methods

### 2.1. Study Design and Participants

This cross-sectional study received ethical approval from the Umm Al-Qura University Institutional Research Board (Approval No. HAPO-02-K-012-2023-09-1759, dated 28 September 2023). The study was conducted from 1 October 2023 to 28 February 2024 in Saudi Arabia. The survey link included the study’s objectives and a consent form for voluntary participation. No identifying or private information was collected, and all responses were kept confidential. The study included parents of children with a reported food allergy under the age of 18 who were residents of Saudi Arabia. Individuals working in the medical field, those who declined participation, and those who did not complete the questionnaire were excluded. By using OpenEpi software version 3, the sample size was calculated, while assuming a population of 3,539,274 (based on a parent-reported food allergy rate of 11% in Saudi Arabia multiplied by the population size based on the 2022 Saudi census of 32,175,224), a 95% confidence interval, and a 20% anticipated frequency rate of EAI use. This resulted in a minimum sample size of 246. The final sample size included 296 parents with reported food-allergic children.

### 2.2. Study Instruments

A self-administered questionnaire to assess food allergies was adapted from previously published instruments [8]. The questionnaire was distributed via social media platforms (X, WhatsApp, Instagram, and Facebook) with the help of 42 data collectors across all regions of Saudi Arabia. Responses were directly exported into Microsoft Excel. The questionnaire was translated into Arabic by two independent bilingual experts with experience in medical translation. Discrepancies between the translated versions were reviewed and resolved by a third bilingual translator. An expert panel, consisting of two bilingual allergists and one bilingual emergency medicine physician, reviewed both the original and translated versions to evaluate content equivalence, clarity, and cultural relevance. The panel approved the final Arabic version. To assess clarity and ensure comprehensibility, a pilot study was conducted with 20 parents from diverse backgrounds. Feedback from this group was used to confirm the lack of ambiguity in the questionnaire items. Of note, we did not conduct full psychometric validation of the questionnaire.

The final questionnaire consisted of three sections: demographic information (child’s age, gender, nationality, parental age, and socioeconomic status), parents’ allergy history and the child’s food allergies (FAs), and parents’ attitudes and practices regarding managing their children’s FAs (Supplementary Material). Informed consent was obtained from all participants.

### 2.3. Statistical Analysis

Data were entered into Microsoft Excel and analyzed using SPSS version 29. GraphPad Prism version 10 was used to generate figures. Descriptive statistics were used to summarize the demographic characteristics of the study participants. Categorical variables were presented as frequencies and percentages. Associations between variables were examined using the chi-square test, with statistical significance set at *p* < 0.05.

## 3. Results

### 3.1. Demographic of the Study Participants

Out of the 2102 participants who completed the questionnaire, 296 (14.1%) reported having a child with a food allergy and were included in this study. The majority of respondents were female parents (70%). Geographically, participants were somewhat evenly spread, with the western region having the highest representation at 33.8%, followed by the central region at 22% (Table 1). The children in the study had a slight male majority (55.4%). The most common allergic comorbidity was asthma (26%), followed by allergic rhinitis (12.8%). Additional demographic details are presented in Table 1.

### 3.2. Food Allergy Symptoms of the Study Participants

**Figure 1:**
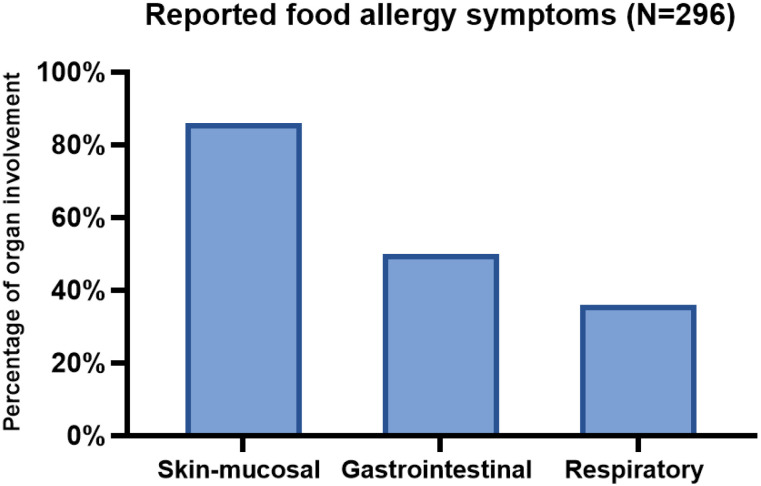
Distribution of food allergy symptoms among children with parent-reported food allergies.

Most children with food allergies presented with cutaneous symptoms (86.1%), followed by gastrointestinal (50.7%) and respiratory symptoms (36.8%) (Figure 1). Approximately 52% of the patients had multiple organ involvement and were presumed to have anaphylaxis based on the clinical criteria proposed at the Second National Institute of Allergy and Infectious Disease/Food Allergy and Anaphylaxis Network (NIAID/FAAN) symposium [22].

**Figure 2:**
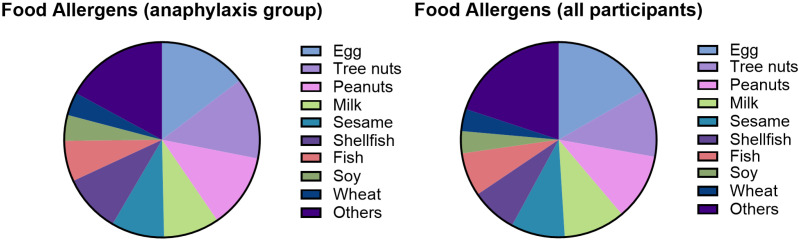
Common food allergens among children with parent-reported food allergies.

The most common food allergens in our study were egg, tree nuts, peanut, milk, and sesame (Figure 2). The list of the most common foods in patients with presumed anaphylaxis was similar to the overall study (Figure 2). Of note, 40% of the study subjects had a single food trigger for their food allergy.

### 3.3. Factors Associated with Epinephrine Auto-Injector Possession

A total of 38.5% parents with food-allergic children were managed by an allergist, 18.2% were managed by a general practitioner, and 43.2% did not follow up with medical providers for their food allergy. Allergy testing confirmed the diagnosis in 42.2% of cases, with skin or blood tests. Only 23.3% of children with a food allergy had an epinephrine auto-injector (EAI) available. To identify factors associated with EAI possession rate, we examined the relationships of demographic factors and possession of injectable epinephrine (Table 2). Parental education significantly influenced EAI use, with higher rates among parents with master’s or doctoral degrees (43%) compared to high school graduates (<4%, *p* < 0.001). Of note, there was no correlation between family income and EAI possession. Interestingly, younger children exhibited a higher EAI possession rate. While no association was found between EAI use and comorbid allergies or paternal allergy history, a significant correlation with maternal allergy history was noted (*p* = 0.02) (Table 2).

### 3.4. Clinical Presentation and EAI Possession

Children with more severe allergic reactions, such as gastrointestinal or respiratory symptoms, were significantly more likely to possess an EAI (*p* < 0.001 and *p* = 0.030, respectively). Additionally, individuals with multiple food allergies demonstrated a higher rate of EAI possession (*p* < 0.001). Children managed by allergists were more likely to possess an EAI (50.9%) compared to those managed by general practitioners (15%) or those without professional medical management (<5%, *p* < 0.001) (Table 3).

## 4. Discussion

This study aimed to assess the rate of epinephrine auto-injector (EAI) possession among Saudi children with parent-reported food allergies and to identify factors associated with EAI use. Our findings revealed a concerningly low EAI possession rate (23.3%), indicating a significant gap in preparedness for managing food allergy-induced anaphylactic reactions in Saudi families.

Consistent with previous studies from Saudi Arabia [8,23,24], our results indicate a high prevalence of parent-reported food allergies among Saudi children (14%). This rate is slightly higher than the reported rate in Brazil (11.7%) and the United State (7.6%) [6,7]. Egg was the most common food allergen, which aligns with findings from other studies conducted in the Arabic gulf region [8,25,26]. In contrast, seafood has been identified as a primary allergen in countries such as Brazil, China, and Korea [5,7,27,28], while peanuts has emerged as the most common allergen in the United States [6,29]. These disparities may be attributed to cultural differences in dietary habits, culinary practices, and the age at which specific foods are introduced into the diet [30].

Asthma was the most common allergic comorbidity in our study, and this is consistent with the literature, which has documented the high prevalence of atopic diseases among patients with food allergies [8,31,32,33,34]. Furthermore, a history of asthma is a risk factor for mortality in food-induced anaphylaxis, with around 70% of individuals who died from food reactions having a history of asthma [35,36].

A food allergy is the most common cause of anaphylaxis in children, with evidence of increasing incidence over time [37,38,39]. The most common clinical presentations of an allergic reaction to food are cutaneous symptoms such as acute urticaria, angioedema, and pruritus, as seen in our studies [40,41,42]. However, systemic involvement, such as hypotension, respiratory, or gastrointestinal symptoms, is required to qualify for the diagnosis of anaphylaxis [12,41]. Although the fatal rate from food-related anaphylaxis is low with an estimated rate to be 0.04 per million per year in the US [43], the delayed use of an EAI was a major risk factor for fatal outcomes [20]. Most of the major allergy professional societies’ guidelines strongly recommend that food-allergic patients carry an EAI [12,40,44,45,46,47].

Despite the important role of the EAI in managing food-induced anaphylaxis, it is not readily available in many countries, especially in the developing world [48,49,50]. In these countries, patients and families need to order EAIs online or travel to purchase them if they can afford the expense, with many families unable to afford EAIs [49,50]. Fortunately, EAIs are available in Saudi Arabia and are covered by the government-run universal healthcare system. However, their availability is inconsistent across the country. For example, EAIs are widely available in the central and western regions, but in other areas—particularly the northern and southern regions—they are typically found only in large tertiary hospitals. This disparity may partially explain the lower EAI possession rates reported in these less-served areas, although the regional differences did not reach statistical significance in our study.

Another barrier to EAI possession is that a prescription is required, which limits access among families who do not receive regular follow-up from healthcare providers. In our cohort, EAI possession was markedly lower among families without medical care (<5%) compared to those whose children were evaluated by allergists, where possession rates exceeded 50%. Based on the authors’ clinical experience, many healthcare providers and patients remain unaware that EAIs are both available and covered within the healthcare system. In our study, the EAI possession rates in Saudi Arabia (23%) are much lower than those reported in the United States [51,52].

In general, there appears to be a significant lack of knowledge about food allergy management among both the general public and school teachers in Saudi Arabia [8,53,54]. There was a higher possession rate in patients presenting with more severe symptoms or with multiple food allergies, which could be partly due to their higher rate of referral to specialists in that population. Further research is needed to confirm this hypothesis. Lastly, there was a relationship between the parents’ level of education and EAI possession. This might reflect a higher parental awareness of the risk of severe reactions or better access to healthcare and specialty care.

While this study provides valuable insights into EAI possession among Saudi children with food allergies, it has some limitations. The cross-sectional design of the study prevents causal inferences, while relying on parental reporting may also introduce recall bias and preclude us from confirming the diagnosis of anaphylaxis, which relies on physical examination as well as patient symptoms. Further research is needed to address the specific barriers to EAI use and to develop effective strategies to increase EAI possession and preparedness.

## 5. Conclusions

In conclusion, our findings revealed a suboptimal rate of EAI possession among Saudi children with food allergies. Targeted educational interventions, increased access to allergists, and comprehensive management plans are essential to improve preparedness for anaphylaxis.

## Figures and Tables

**Table 1:** Demographic characteristics of the study participants (N = 296).

Characteristic		Frequency	Percentage
Parent’s gender	Female	206	69.6%
	Male	90	30.4%
Parent’s age	Less than 26 years	23	7.8%
	27–35 years	74	25.0%
	36–45 years	119	40.2%
	46–55 years	77	26.0%
	More than 55 years	3	1.0%
Region	Western region	100	33.8%
	Eastern region	45	15.2%
	Central region	65	22.0%
	Southern region	42	14.2%
	Northern region	44	14.9%
Educational level	High school level or lower	51	17.2%
	Bachelor’s degree	206	69.6%
	Master’s; PhD or equivalent	39	13.2%
Family monthly income	Less than 5000 SR *	94	31.8%
	6000 SR–10,000 SR *	91	30.7%
	More than 10,000 SR *	111	37.5%
Gender of the child	Female	132	44.6%
	Male	164	55.4%
Child’s age	5 years and below	78	26.4%
	6 years–10 years	103	34.8%
	11 years–18 years	105	35.5%
Allergic comorbidity	Asthma	79	26.7%
	Allergic rhinitis	38	12.8%
	Drug allergy	9	3.0%

* “SR” refers to Saudi Riyals.

**Table 2:** The impact of demographic factors on epinephrine auto-injector (EAI) possession.

Characteristic		FA Children Without EAI (N = 227)	FA Children with EAI (N = 69)	*p*-Value
Parent’s gender	Female	162 (71.4%)	44 (63.8%)	0.230
	Male	65 (28.6%)	25 (36.2%)	
Parent’s age	Less than 26 years	17 (7.5%)	6 (8.7%)	0.047
	27–35 years	52 (22.9%)	22 (31.9%)	
	36–45 years	90 (39.6%)	29 (42.0%)	0.007
	46–55 years	67 (29.5%)	10 (14.5%)	
	More than 55 years	1 (0.4%)	2 (2.9%)	
Region	Western region	72 (31.7%)	28 (40.6%)	0.069
	Eastern region	35 (15.4%)	10 (14.5%)	
	Central region	45 (19.8%)	20 (29.0%)	
	Southern region	36 (15.9%)	6 (8.7%)	
	Northern region	39 (17.2%)	5 (7.2%)	
Educational level	High school level or lower	49 (21.6%)	2 (2.9%)	<0.001
	Bachelor’s degree	156 (68.7%)	50 (72.5%)	
	Master’s; PhD or equivalent	22 (9.7%)	17 (24.6%)	
Family monthly income	Less than 5000 SR	76 (33.5%)	18 (26.1%)	0.308
	6000 SR–10,000 SR	65 (28.6%)	26 (37.7%)	
	More than 10,000 SR	86 (37.9%)	25 (36.2%)	
Gender of the child	Female	108 (47.6%)	24 (34.8%)	
	Male	119 (52.4%)	45 (65.2%)	
Child’s age	5 years and below	54 (23.8%)	24 (34.8%)	0.008
	6 years–10 years	74 (32.6%)	29 (42.0%)	
	11 years–18 years	91 (40.1%)	14 (20.3%)	
Comorbid allergies 1	Absent	145 (63.9%)	39 (56.5%)	0.270
	Present	82 (36.1%)	30 (43.5%)	
Father’s allergies 1	Absent	106 (46.7%)	30 (43.5%)	0.639
	Present	121 (53.3%)	39 (56.5%)	
Mother’s allergies 1	Absent	108 (47.6%)	22 (31.9%)	0.021
	Present	119 (52.4%)	47 (68.1%)	

^1^ “Allergies” encompass any of the following conditions: asthma, allergic rhinitis, food allergies, or drug allergies.

**Table 3:** Clinical presentation in children with food allergies (FAs) by epinephrine auto-injector (EAI) possession.

Characteristic		FA Children Without EAI (N = 227)	FA Children with EAI (N = 69)	*p*-Value
Cutaneous symptoms	Absent	30 (13.2%)	11 (15.9%)	0.566
	Present	197 (86.8%)	58 (84.1%)	
Gastrointestinal symptoms	Absent	125 (55.1%)	21 (30.4%)	<0.001
	Present	102 (44.9%)	48 (69.6%)	
Respiratory symptoms	Absent	151 (66.5%)	36 (52.2%)	0.030
	Present	76 (33.5%)	33 (47.8%)	
Presumed anaphylaxis	Absent	117 (51.5%)	25 (36.2%)	0.026
	Present	110 (48.5%)	44 (63.8%)	
Multiple food allergens	Absent	103 (45.4%)	15 (21.7%)	<0.001
	Present	124 (54.6%)	54 (78.3%)	
History of positive skin or specific IgE allergy test	Absent	165 (72.7%)	6 (8.7%)	<0.001
	Present	62 (27.3%)	63 (91.3%)	
Diagnosing physician	Subspecialist	56 (24.7%)	58 (84.1%)	<0.001
	General physician	46 (20.3%)	8 (11.6%)	
	Self-diagnosed	125 (55.1%)	3 (4.3%)	

## Data Availability

The raw data that support the findings of this study are available from the corresponding author, [AK], upon reasonable request.

## Supplementary Materials

The following supporting information can be downloaded at https://www.mdpi.com/article/10.3390/jcm14155274/s1. Section S1: Demographic information. Section S2: Food allergies and related conditions. Section S3: Management of your child’s food allergy.
