# Supplementary material for: Possession of Injectable Epinephrine Among Children with Parent-Reported Food Allergies in Saudi Arabia
Source: J Clin Med. 2025 Jul 25;14(15):5274. doi: 10.3390/jcm14155274 (PMC12347483; doi:10.3390/jcm14155274)
Supplement: Supplementary file 1 [file jcm-14-05274-s001.zip › jcm-3643229-supplementary.pdf]

First Section:

Section S1: Demographic

Information

1. What is your gender?

- Male
- Female

2. What is your age?

- Less than 26 years
- 27–35 years
- 36–45 years
- 46–55 years
- More than 55 years

3. In which region do you currently live?

- Western Region
- Eastern Region
- Central Region
- Southern Region
- Northern Region

4. What is the highest level of education you have completed?

- High school or lower
- Bachelor’s degree
- Master’s degree, PhD, or equivalent

5. What is your family's monthly income?

- Less than 5,000 SAR
- 6,000–10,000 SAR
- More than 10,000 SAR

6. Do you have children younger than 18 years old?

- Yes
- No

7. If yes, what is the age of your child?
   Child's age: _______ years
8. What is the gender of your child?

- Male
- Female

Section S2: Food Allergies and Related Conditions

9. Does your child suffer from food allergies?

- Yes
- No

10. Does the father have any of the following allergic conditions? *(Select all that apply)*

- Asthma
- Allergic rhinitis
- Food allergy
- Drug allergy
- None of the above

11. Does the mother have any of the following allergic conditions? *(Select all that apply)*

- Asthma
- Allergic rhinitis
- Food allergy
- Drug allergy
- None of the above

12. Does your child suffer from any of the following allergic conditions? *(Select all that apply)*

- Asthma

- Allergic rhinitis
- Drug allergy
- None of the above

13. If your child has a food allergy, which of the following foods trigger allergic reactions? *(You can select more than one)*

- Egg
- Peanuts
- Sesame
- Shellfish (shrimp, oysters, king crab, mussels, lobster, octopus)
- Milk
- Fish
- Soybean
- Tree nuts (pistachios, cashews, almonds, hazelnuts, walnut, pecan etc.)
- Wheat
- Other (please specify): _____________

14. What symptoms does your child usually experience during a food allergy reaction (for each food) ? *(You can select more than one)*

- Urticarial rash (hives)
- Vomiting
- Diarrhea
- Abdominal pain
- Shortness of breath
- Itching without rash
- Swelling of lips and/or tongue
- Other (please specify): _____________

Section S3: Management of Your Child's Food Allergy

15. Do you follow up with a doctor for your child's food allergy?

- Yes, with an allergist
- Yes, with a general practitioner
- No, we do not follow up

16. Has your child undergone allergy testing (skin prick test or blood test) to confirm the diagnosis?

- Yes, and the test was positive
- Yes, and the test was negative
- No, the test was not performed

17. Do you have a prescription for an epinephrine auto-injector (EpiPen) for your child?

- Yes
- No

18. Which of the following symptoms would lead you to administer an EpiPen to your child? *(You can select more than one)*

- Difficulty swallowing
- Swelling of the tongue
- Difficulty talking
- Bluish skin color (cyanosis)
- Shortness of breath
- Loss of consciousness
- Noisy breathing
- Repetitive or hacking cough
- Hoarseness of voice
- Hives on the body
- Swelling around the eyes
- Itching of the hands or feet

- Skin flushing
- Sweating
